# Extracts of *Tectona grandis* and *Vernonia amygdalina* have anti-*Toxoplasma* and pro-inflammatory properties *in vitro*

**DOI:** 10.1051/parasite/2018014

**Published:** 2018-03-13

**Authors:** Mlatovi Dégbé, Françoise Debierre-Grockiego, Amivi Tété-Bénissan, Héloïse Débare, Kodjo Aklikokou, Isabelle Dimier-Poisson, Messanvi Gbeassor

**Affiliations:** 1 Laboratoire de Physiologie et de Pharmacologie des Substances Naturelles, Faculté des Sciences, Université de Lomé, B.P. 1515, Lomé 01 Togo; 2 ISP, INRA, Université Tours, 37380 Nouzilly France

**Keywords:** *T. grandis*, *V. amygdalina*, cytotoxicity, oxidation, inflammation

## Abstract

*Tectona grandis* (teak) and *Vernonia amygdalina* (bitter leaf) are plants used in traditional medicine in West Africa. In this study, we tested ethanolic and hydro-ethanolic extracts of bark and leaves of *T. grandis* and ethanolic extract of leaves of *V. amygdalina* for their inhibitory effect on *Toxoplasma gondii*, a protozoan parasite responsible for toxoplasmosis. Ethanolic extract of *V. amygdalina* leaves had proportional contents of phenols, tannins, flavonoids, and polysaccharides. This extract presented the highest efficacy against *T. gondii*, the lowest cytotoxicity to mammalian cells, but moderate anti-oxidant activity compared to other plant extracts. Ethanolic extract of *T. grandis* bark also had elevated anti-*T. gondii* activity, low cytotoxicity on mammalian cells, and one of the highest anti-oxidant activities. However, the phytochemical content of this extract was not very different from the hydro-ethanolic extract, which had no anti-*T. gondii* activity. In addition, ethanolic extract of *V. amygdalina* leaves, but not of *T. grandis* bark, significantly increased the production of TNF-α and NO by antigen-presenting cells. Both extracts had the tendency to decrease expression of major histocompatibility complex molecules at the surface of antigen-presenting cells, while they did not modulate the percentage of apoptotic cells. A study of signalling pathways would help to determine the mechanisms of action of these plant extracts.

## Abstract (fr)

*Tectona grandis* (teck) et *Vernonia amygdalina* sont des plantes utilisées dans la médecine traditionnelle en Afrique de l’Ouest. Dans cette étude, l’effet inhibiteur d’extraits éthanoliques et hydro-éthanoliques d’écorce et de feuilles de *T. grandis* et de l’extrait éthanolique des feuilles de *V. amygdalina* a été étudié sur *Toxoplasma gondii*, un parasite protozoaire responsable de la toxoplasmose. L’extrait éthanolique des feuilles de *V. amygdalina* avait des quantités équivalentes de phénols, tanins, flavonoïdes et polysaccharides. Cet extrait présentait la plus grande efficacité contre *T. gondii*, la plus faible cytotoxicité vis-à-vis de cellules de mammifères, mais une activité anti-oxydante moyenne comparée aux autres extraits de plantes. L’extrait éthanolique d’écorce de *T. grandis* avait aussi une activité anti-*T. gondii* élevée, une faible cytotoxicité vis-à-vis des cellules de mammifères et l’une des activités anti-oxydantes les plus élevées. Cependant, le contenu phytochimique de cet extrait n’était pas très différent de l’extrait hydro-éthanolique qui n’avait pas d’activité anti-*T. gondii*. De plus, l’extrait éthanolique des feuilles de *V. amygdalina*, mais pas de l’écorce de *T. grandis*, augmentait significativement la production de TNF-α et de NO par les cellules présentatrices d’antigènes. Les deux extraits avaient tendance à diminuer l’expression des molécules du complexe majeur d’histocompatibilité à la surface des cellules présentatrices d’antigènes alors qu’ils ne modulaient pas le pourcentage de cellules apoptotiques. L’étude des voies de signalisation permettrait de comprendre les mécanismes d’action de ces extraits de plantes.

## Introduction

*Toxoplasma gondii* is a protozoan parasite belonging to the Phylum Apicomplexa like *Plasmodium*, the causal agent of malaria. It is estimated that one third of the world’s population is infected with *T. gondii*. Toxoplasmosis is generally asymptomatic in immune-competent individuals but the persistence of the parasite in cysts located in the brain, muscles and eyes may lead to irreversible damage in case of immune-depression. More importantly, *T. gondii* is able to cross the placental barrier during pregnancy to infect the foetus. Congenital toxoplasmosis can cause malformations (calcification, hydrocephaly, etc.) or abortion. Toxoplasmosis is considered as a neglected disease worldwide and in the United States of America [32,12]. In this country, *T. gondii* is the second leading cause of deaths attributable to food-borne illness [5] and toxoplasmosis is one of the five neglected parasitic infections targeted for public health action. Current treatments for toxoplasmosis are the combination of 2,4-diaminopyrimidines (pyrimethamine, trimethoprim) and sulfamides (sulfadiazine, sulfamethoxazole) [20]. Alternative treatments (clindamycin, atovaquone) may be indicated in cases of intolerance to first-line treatments. Since pyrimethamine is potentially teratogenic, spiramycin is administered during the first trimester of pregnancy. Furthermore, 2,4-diaminopyrimidines induce reversible depression of the bone marrow prevented by folinic acid supplementation given concomitantly with the treatment. Due to the lack of specificity and to the limited efficacy of these current treatments, there is a need for new compounds to cure toxoplasmosis with less toxicity and short treatment courses. Several research groups are working on the development of new synthetic compounds against *T. gondii* (for a review of the last ten years, see reference [19]). Only the bumped kinase inhibitor (BKI)-1294 has been tested in a mouse model of congenital toxoplasmosis with more than 90% reduction in congenital infection [21]. Unfortunately, BKI-1294 seems to have a detrimental effect on fertility. Most of the new drugs approved to treat infectious diseases originate from natural products. For this reason, anti-*Toxoplasma* activities are screened for in extracts of plants used in traditional medicine in West Africa, and have shown highly variable efficacy *in vitro* (for review, see reference [29]). In the present work, we studied the anti-*Toxoplasma* effect of ethanolic and hydro-ethanolic extracts of bark and leaves of *Tectona grandis* (Lamiaceae), the well-known teakwood used as a material for furniture, and of ethanolic extract of leaves of *Vernonia amygdalina* (Asteraceae) or bitter leaf, a plant known for its production of beneficial secondary metabolites. This nontoxic plant, consumed as a vegetable, is used in traditional folk medicine to treat or control more than twenty diseases including malaria [31], while *T. grandis* is mainly used in skin diseases as an antiseptic, wound-healing and anti-acne agent. Nevertheless, methanolic extract of *T. grandis* bark had leishmanicidal activity on *L. major* promastigotes [30] and methanolic extract of *T. grandis* leaves was highly effective against multidrug-resistant uropathogenic bacteria *in vitro* [17]. In the present work, the anti-oxidant, anti-haemolytic, and pro-inflammatory properties of plant extracts were also studied.

## Materials and methods

### Compliance with the Nagoya Protocol

Togo signed the Nagoya Protocol in 2011 and is party since 2016. We received authorisation from the *Institut Togolais de Recherche Agronomique* (ITRA) to collect samples and to use plant extracts at the University of Tours (France) for research experiments.

### Chemicals

Ascorbic acid, 2,2’-azobis 2 amidinopropane dihydrochloride (AAPH), 2,2-diphenyl*-*1-picrylhydrazyl (DPPH), dimethyl sulfoxide (DMSO), hydrogen peroxide (H_2_O_2_), gallic acid, glucose, and rutin were purchased from Sigma-Aldrich Chemicals. All solvents of analytical grade were purchased from Merck Limited.

### Plant collection, identification, and taxonomy

Samples of *Tectona grandis* L.f., 1782 (Verbenaceae) and *Vernonia amygdalina* Del. (Asteraceae), both plants freely available in Togo, were collected in November 2014. The leaves and barks of *Tectona grandis* were harvested from the experimental farm of the University of Lomé. The leaves of *Vernonia amygdalina* were harvested in the village of Sika-Kondji, Yoto district of Togo (6°37'60" N and 1°34'60" E). Preliminary identification of the plants was done in the field. Afterwards, herbarium specimens were prepared and photographs were taken to aid in the confirmation of the identity of the plants. They were identified and authenticated by comparison with available voucher specimens in the herbarium section of the Laboratory of Botany and Plant Ecology of the University of Lomé (Togo), using taxonomic keys of online databases of PROTA (PROTA4U, Plant resources of Tropical Africa) on the website: www.prota.org. The nomenclature of species was checked using the online database of the International Plant Names Index (IPNI) (http://www.ipni.org/ipni/plantnamesearchpage.do)

- Leaves and bark of *Tectona grandis* voucher specimen *Tectona grandis*: TOGO15318

- Leaves of *Vernonia amygdalina* voucher specimen *Vernonia amygdalina*: TOGO15314.

### Phytochemical screening

The materials were washed in water to remove dirt and unwanted particles. Washed materials were dried at room temperature for several days. Then ethanolic and hydro-ethanolic extractions were performed by maceration of plant samples in the solvent (ethanol or mixture of ethanol and distilled water v/v) and with periodic agitation for 72 hours. Afterwards, solvent was evaporated under reduced pressure with a rotary evaporator and the extracts were lyophilised. Yield of dried extract was 7.4% and 7.5% for ethanolic and hydro-ethanolic extracts of *T. grandis* leaves, 11.1% and 12.4% for ethanolic and hydro-ethanolic extracts of *T. grandis* bark, and 9.6% for ethanolic extract of *V. amygdalina* leaves (percentage weight of the starting dried plant material).

Analysis of phytochemical contents was done on lyophilised extracts of leaves and bark of *T. grandis* and on lyophilised extract of leaves of *V. amygdalina* suspended in methanol (at 1 mg/mL). Total phenols were quantified by spectrophotometry using the Folin-Ciocalteu method with gallic acid as standard. Total flavonoids were quantified by spectrophotometry using the aluminium chloride colorimetric method with rutin as the standard curve. Polysaccharides were quantified according to Dubois *et al*. [11] with glucose as the standard curve.

### Anti-oxidant activity

The free radical scavenging activity of the plant extracts was measured by the DPPH (2,2-diphenyl-1-picrylhydrazyl) and the AAPH (2,2’-azobis 2 amidinopropanedihydrochloride) radical scavenging activity assays. For the first method, plant extracts were prepared in methanol. One volume of 0.1 mM DPPH was mixed with one volume of the plant extracts at different concentrations, ascorbic acid as positive control, or methanol (blank). Absorbance was read at 480 nm after 30 min incubation at room temperature in the dark. The percentage of DPPH free radicals scavenging was calculated as ([Optical Density blank/O.D. plant extract]/O.D. blank) x100. In the second method, the addition of the peroxyl radical initiator AAPH to erythrocytes induces their haemolysis. One volume of sheep blood cell suspension (from the *Institut National de la Recherche Agronomique* Centre Val-de-Loire) was mixed with one volume of PBS, one volume of 200 mM AAPH, and one volume of the plant extracts at different concentrations or PBS (complete lysis), or ascorbic acid (no lysis). The mixture was incubated at 37 °C for 3 hours, washed with PBS, and centrifuged at 1000 *g* for 10 min. Absorbance of the supernatant was read at 565 nm and percentage of haemolysis inhibition was calculated as ([O.D. PBS/O.D. plant extract]/O.D. PBS) x100.

### *In vitro* inhibition of *T. gondii*

Plant efficacy on *T. gondii* was tested in a colometric assay, as previously described [18]. For this, human foreskin fibroblasts (HFF, ATCC number CRL-1634) were seeded in 96-well plates at 2 × 10^4^ cells per well in Dulbecco’s Modified Eagle Medium (DMEM without phenol red, Pan Biotech GmbH) with 1% fetal calf serum and 2 mM glutamine at 37 °C in 5% CO_2_ atmosphere. Ethanolic extracts of *V. amygdalina* leaves (Va-LE), hydro-ethanolic and ethanolic extracts of *T. grandis* leaves (Tg-LH and Tg-LE), hydro-ethanolic and ethanolic extracts of *T. grandis* bark (Tg-BH and Tg-BE) were lyophilised, solubilised in DMSO (stock solution at 250 mg/mL), and diluted in DMEM without phenol red. After 24 h, *T. gondii* parasites (100 tachyzoites of the *T. gondii* RH-β-gal strain carrying the *E. coli lacZ* gene coding for β-galactosidase under the control of the *T. gondii* *sag1* promoter) and plant extracts (1 − 200 μg/mL in quadruplicate) were added to host cells at the same time. In negative control wells, tachyzoites were incubated with same amounts of DMSO alone with final maximum amount of 0.02%. Plant extracts were maintained throughout the parasite invasion and proliferation steps. After 96 h incubation at 37 °C in 5% CO_2_ atmosphere, release of the β-galactosidase was achieved with lysis solution (Triton X-100 at 0.1%, Sigma) and chlorophenol red-β-D-galactopyranoside (Sigma) was added at 1 mM in 100 mM HEPES pH 8. Optical density of the red colouration was measured at 565 nm. The IC_50_ (50% inhibitory concentration) was calculated from the β-galactosidase activity in the presence of plant extracts compared to non-treated parasites.

### Cytotoxicity assay

HFFs (2 × 10^4^ cells) were cultivated in 96-well plates in 150 μL DMEM with 1% FCS, 2 mM glutamine and the plant extracts at different concentrations, in triplicate. Plates were incubated at 37 °C in a 5% CO_2_ atmosphere for 96 h. UptiBlue^TM^ (15 μL, Interchim) was then added for 4 h incubation in the dark at 37 °C in a 5% CO_2_ atmosphere. O.D. was measured at 565 nm and 630 nm to calculate the reduction of the oxidation-reduction indicator. CC_50_ (50% cytotoxic concentration) was calculated from the reduction in the presence of plant extracts compared to non-treated cells.

### Cell stimulation and tumour necrosis factor (TNF)-α/nitric oxide (NO) quantification

Cells were seeded at 2 × 10^5^ in 96-well plates in 100 μL DMEM for the RAW 264.7 macrophage cell line (ATCC^®^ TIB-71^TM^), at 10^6^ in 24-well plates in 300 μL DMEM for peritoneal exudate cells (PECs) harvested from Swiss OF1 mice by washing with 5 mL DMEM (directive 2010/63/EU not applied [chapter 1, article 1.5.]), and at 10^6^ in 24-well plates in 300 μL RPMI for the SRDC dendritic cell line [28]. The cells were stimulated at 37 °C in 5% CO_2_ atmosphere for 24 h with plant extracts at different concentrations in 100 or 300 μL medium. DMSO at the lowest dilution was used as a negative control. Levels of tumour necrosis factor (TNF)-α were quantified in the cell culture supernatants of RAW 264.7 and SRDC by using specific sandwich enzyme-linked immunosorbent assay (ELISA) from Affymetrix eBioscience. Levels of nitric oxide (NO) were measured with the Griess assay. For this, two volumes of culture supernatant were mixed with one volume of 0.1% naphthyl ethylenediamine (in water) and one volume of 1% sulfanilamide (in 5% phosphoric acid). A solution of NaNO_2_ was used as standard. Absorbance was read at 540 nm.

### Major histocompatibility complex (MHC) expression on SRDC and apoptosis of PECs

After stimulation, SRDCs and PECs were detached with accutase (Affymetrix eBioscience). SRDCs were centrifuged for 5 min at 300 *g* and saturated for 30 min on ice in PBS containing 1% bovine serum albumin, 2% mouse serum, and 0.1% azide (PBS-BSA-azide). After centrifugation, 3 × 10^5^ SRDCs were incubated for 30 min on ice in the dark in PBS-BSA-azide with 0.5 μg FITC mouse anti-mouse H-2K[k] MHC class I antibody (clone 36-7-5, BD Pharmingen^TM^), FITC mouse anti-mouse I-E[k] MHC class II antibody (clone 14-4-4S), or FITC mouse IgG2a, κ isotype control (clone G155-178). After centrifugation, SRDCs were suspended in 300 μL PBS and analysed by flow cytometry (BD FACSCalibur^TM^ and CellQuest^TM^ software, BD Bioscience). PECs were incubated for 15 min at 4 °C in the dark in 100 μL binding buffer (10 mM Hepes pH 7.4, 140 mM NaCl, 5 mM CaCl_2_) with 5 μL FITC Annexin V (BD Pharmingen^TM^) and 5 μg/mL propidium iodide (Sigma). After centrifugation, PECs were suspended in 300 μL binding buffer and analysed by flow cytometry.

### Statistical analysis

The non-parametric Kruskal–Wallis test followed by the Dunn’s multiple comparison test was used for statistical evaluation, and a *p* value < 0.05 was considered significant (GraphPad Prism 5 software).

## Results and discussion

### Phytochemical screening of the plant extracts

The phytochemical analysis was performed on the lyophilised extracts of leaves and bark of *T. grandis* and on the lyophilised extract of leaves of *V. amygdalina* for identification of the constituents. Contents of phytochemicals of bark and leaves of *T. grandis* are not particularly different (Tab. 1). Higher amounts of all phytochemicals were measured in ethanolic extract (Tg-BE) than in hydro-ethanolic extract (Tg-BH) of the bark of *T. grandis*. The ethanolic extract of the leaves of *T. grandis* (Tg-LE) had the highest concentration of phenols and flavonoids, while the hydro-ethanolic extract of the leaves of *T. grandis* (Tg-LH) had the highest concentrations of tannins and polysaccharides. The hydro-ethanolic extract of the bark of *T. grandis* (Tg-BH) also had high levels of polysaccharides and intermediate levels of phenols, tannins, and flavonoids. The leaves of *V. amygdalina* had less phenols, flavonoids, and polysaccharides than the leaves of *T. grandis*. Anyasor *et al.* [3] have found saponins, phenols, flavonoids and alkaloids in methanolic extract of leaves of *V. amygdalina* from South-Western Nigeria, while aqueous extract contained in addition tannins, phlobatannins and cardiotonic glycosides. A recent study has shown that leaves of *V. amygdalina* contain alkaloids, tannins, flavonoids, saponin, anthraquinones, and cardiotonic glycosides, and demonstrated their safety after survival of all rats that received 3200 mg/kg of the extract *per os* [2].

**Table 1:** Phytochemical screening of the plant extracts.

	Phenols (μg/mL)	Tannins (μg/mL)	Flavonoids (μg/mL)	Polysaccharides (μg/mL)
	(n = 7)	(n = 4)	(n = 14)	(n = 14)
Tg-BE	153 ± 10	103 ± 15	135 ± 51	430 ± 32
Tg-BH	147 ± 12	93 ± 5	131 ± 49	396 ± 46
Tg-LE	**206 ±** **18**	101 ± 4	**183 ±** **69**	386 ± 48
Tg-LH	141 ± 44	**122 ±** **4**	124 ± 49	**440 ±** **57**
Va-LE	161 ± 9	118 ± 8	141 ± 54	253 ± 45

Values are means ± S.D.

Tg-BE: *T. grandis* − bark ethanolic extract; Tg-BH: *T. grandis* − bark hydro-ethanolic extract; Tg-LE: *T. grandis* − leaf ethanolic extract; Tg-LH: *T. grandis* − leaf hydro-ethanolic extract; Va-LE: *V. amygdalina* − leaf ethanolic extract.

### Anti-*Toxoplasma* efficacy and cytotoxicity induced by the plant extracts

The plant extracts were tested for their anti-*Toxoplasma* efficacy *in vitro*. The concentration inducing 50% reduction in parasite number (IC_50_) was highly variable from one experiment to another, and the standard error of the mean (S.E.M.) was high (Tab. 2). In our conditions, the ethanolic extract of the leaves of *V. amygdalina* (Va-LE) was the most effective against *T. gondii* with an IC_50_ of 5.6 ± 0.3 μg/mL. Furthermore, this extract was the lowest cytotoxic on the host cells (HFF) with a CC_50_ of 303.6 ± 119.1 μg/mL (selectivity index [CC_50_/IC_50_] of 50). Aqueous extracts of a plant of the same genus, *V. colorata*, also showed growth inhibition of *T. gondii* with IC_50_ values of 17-18 μg/mL and with a toxic effect on MRC5 cells at concentrations > 250 μg/mL [6]. When extracts of *V. colorata* were prepared in organic solvents (ethanol, acetone, dichloromethane), a 5- to 10-fold increase in activity was observed. We have not compared hydro-ethanolic and ethanolic extracts of *V. amygdalina* leaves but the results presented by Omoregie *et al.* [25] have shown that an ethanolic extract of the leaves of this plant had higher anti-*Plasmodium* activity than hydro-ethanolic and aqueous extracts, with a selectivity index of 6. In a more recent study, treatment of mice with aqueous but not ethanolic extract of *V. amygdalina* leaves reduced the *P. berghei* macrogametocyte density by about 50% [1]. These different studies indicate that the anti-parasitic activities of *V. amygdalina* depend on the type of extract and on the targeted parasite. Compared to all other extracts studied here, the ethanolic extract of the leaves of *V. amygdalina* contained the lowest amount of polysaccharides. The proportion of phenols, tannins, flavonoids, and polysaccharides may underlie the *in vitro* effects of *V. amygdalina* leaves extract. Concerning *T. grandis*, the ethanolic extract of bark (Tg-BE) gave the best results in terms of cytotoxicity and anti-*T. gondii* activity, with a selectivity index of 10, while the ethanolic extract of the leaves (Tg-LE) yielded the poorest results, with a selectivity index of 0.3. Hydro-ethanolic extract of both bark (Tg-BH) and leaves (Tg-LH) presented lower cytotoxicity than the ethanolic extract of the leaves but also lower anti-*T. gondii* activities than the ethanolic extract of the bark. There are very few recent data in the literature on the anti-infectious properties of *T. grandis*. Juglone (5-hydroxy-1,4-naphthalenedione) has been identified as a compound with antibacterial activity against *Listeria monocytogenes* and *Staphylococcus aureus* present in methanol extract of *T. grandis* bark [23]. A study investigated the antimicrobial properties of isolated constituents of the leaves of *T. grandis* [22]. Rutin (flavonoid) and ellagic acid (phenol) showed significant activity against *Bacillus subtilis*, *Escherichia coli*, *Klebsiella pneumonia*, and *Staphylococcus aureus*. Gallic acid (phenol) and sitosterol exhibited activity against these bacteria except *S. aureus* and *E. coli*, respectively. Quercitin (flavonoid) showed activity only against *K. pneumoniae* and *S. aureus*. The levels of phenols and flavonoids in the ethanolic extract of the bark of *T. grandis* are similar to those of hydro-ethanolic extract of bark and leaves and may not explain its properties.

**Table 2:** Cytotoxicity on HFF and anti-*Toxoplasma* efficacy of the plant extracts.

	CC_50_ (μg/mL)	IC_50_ (μg/mL)	SI
Tg-BE	147.7 ± 10.9	15.3 ± 0.6	9.9
Tg-BH	85.2 ± 11.7	176.7 ± 76.7	0.5
Tg-LE	38.4 ± 7.8	143.3 ± 35.4	0.3
Tg-LH	45.9 ± 20.1	59.8 ± 0.9	0.8
Va-LE	**303.6 ±** **119.1**	**5.6 ±** **0.3**	**50.7**

Values are means ± S.E.M. of three independent experiments.

Tg-BE: *T. grandis* − bark ethanolic extract; Tg-BH: *T. grandis* − bark hydro-ethanolic extract; Tg-LE: *T. grandis* − leaf ethanolic extract; Tg-LH: *T. grandis* − leaf hydro-ethanolic extract; Va-LE: *V. amygdalina* − leaf ethanolic extract. CC_50_, 50% cytotoxic concentration on HFF; IC_50_, 50% inhibitory concentration on *T. gondii* growth; SI, selectivity index.

### Anti-oxidant properties of the plant extracts

**Figure 1:**
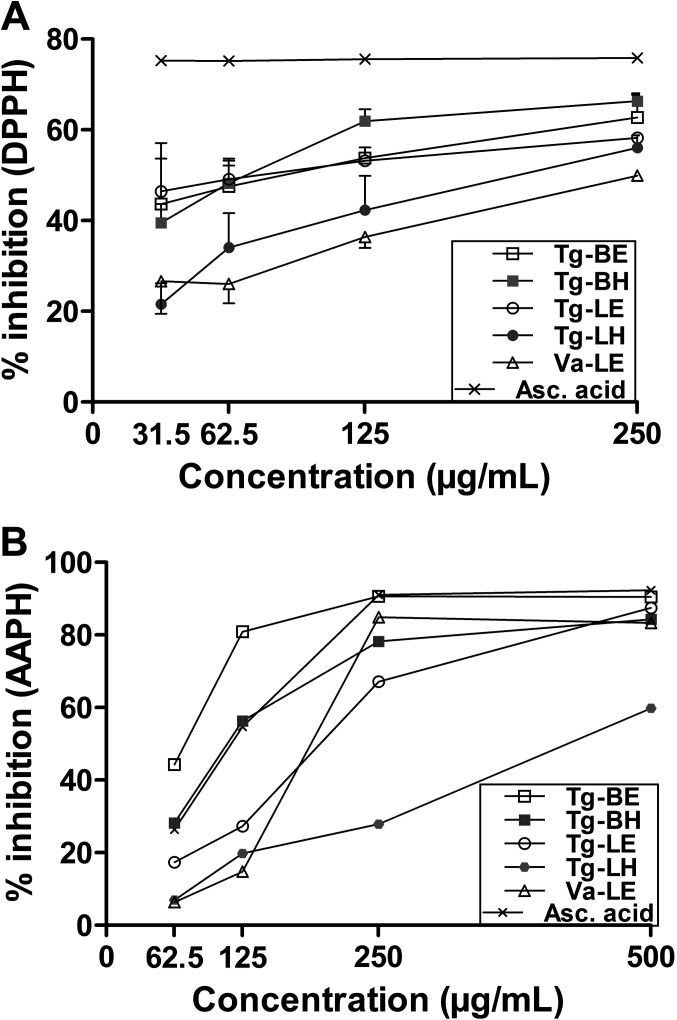
Anti-oxidant activity of the plant extracts evaluated by the DPPH (A) and the AAPH (B) assays. Values of the DPPH assay are means + or − S.D. of three independent experiments. Asc. acid: ascorbic acid; Tg-BE: *T. grandis* − bark ethanolic extract; Tg-BH: *T. grandis* − bark hydro-ethanolic extract; Tg-LE: *T. grandis* − leaf ethanolic extract; Tg-LH: *T. grandis* − leaf hydro-ethanolic extract; Va-LE: *V. amygdalina* − leaf ethanolic extract.

The anti-oxidant activities of the plant extracts were evaluated by the widely used method of electron transfer from anti-oxidant to DPPH (Fig. 1A). Ascorbic acid, known for its anti-oxidant effect, induced 75% inhibition of oxidation at all concentrations tested (31.5 to 250 μg/mL). All plant extracts presented a dose-dependent anti-oxidant effect but with lower efficacy than ascorbic acid, even though the differences are not statistically significant. At the lowest concentrations (31.5 and 62.5 μg/mL), ethanolic extracts of the leaves (Tg-LE) and the bark (Tg-BE) of *T. grandis* were the most effective. At higher concentrations (125 and 250 μg/mL), hydro-ethanolic and ethanolic extracts of *T. grandis* bark (Tg-BH and Tg-BE) had the highest anti-oxidant effects. The ethanolic extract of *V. amygdalina* (Va-LE) and the hydro-ethanolic extract of *T. grandis* leaves (Tg-LH) were the least effective at any concentration tested. A second method was used to study the anti-oxidant properties of the extracts. The addition of the peroxyl radical initiator AAPH to erythrocytes causes oxidation of lipids and proteins in the cell membrane and induces haemolysis, inhibited by anti-oxidant molecules (Fig. 1B). All preparations, even ascorbic acid, should be added at high concentrations to inhibit lysis of erythrocytes. In these conditions, ethanolic and hydro-ethanolic extracts of bark of *T. grandis* (Tg-BE and Tg-BH) were more efficient than ascorbic acid at 62.5 and 125 μg/mL. At the concentration of 250 μg/mL, only ethanolic extract of *T. grandis* bark (Tg-BE) was as effective as ascorbic acid, while ethanolic and hydro-ethanolic extracts of leaves of *T. grandis* (Tg-LE and Tg-LH) presented the lowest activity. At 500 μg/mL, all extracts except Tg-LH almost reached the percentage of oxidation inhibition of ascorbic acid. These results indicate that anti-oxidant activity demonstrated with an artificial method such as DPPH assay might be slightly different in an assay more similar to natural conditions like in the presence of erythrocytes. Our results on the anti-oxidant activity of the ethanolic extract of *T. grandis* leaves are consistent with the study of Diallo *et al*. [8]. The authors induced a model mimicking haemolytic anaemia due to *Plasmodium falciparum* by injecting phenyl hydrazine at 40 mg/kg for 2 days to wistar rats. Ethanolic extract of *T. grandis* leaves administered at 1 g/kg by the oral route for 5 days inhibited anaemia by increasing osmotic resistance of erythrocytes and concentration of haemoglobin [8]. It has been shown that the anti-oxidant activity of the constituents of *T. grandis* leaves depends on the number and the position of hydroxyl groups with the highest activity for the flavonoids quercitin and rutin, followed by the phenols gallic and ellagic acids [22]. Polysaccharides of *T. grandis* bark have also demonstrated anti-oxidant activity with IC_50_ values from 0.1 to 2.4 mg/mL for scavenging of free radical, hydroxyl radical, and NO [27].

### Modulation of antigen-presenting cells by plant extracts

**Figure 2:**
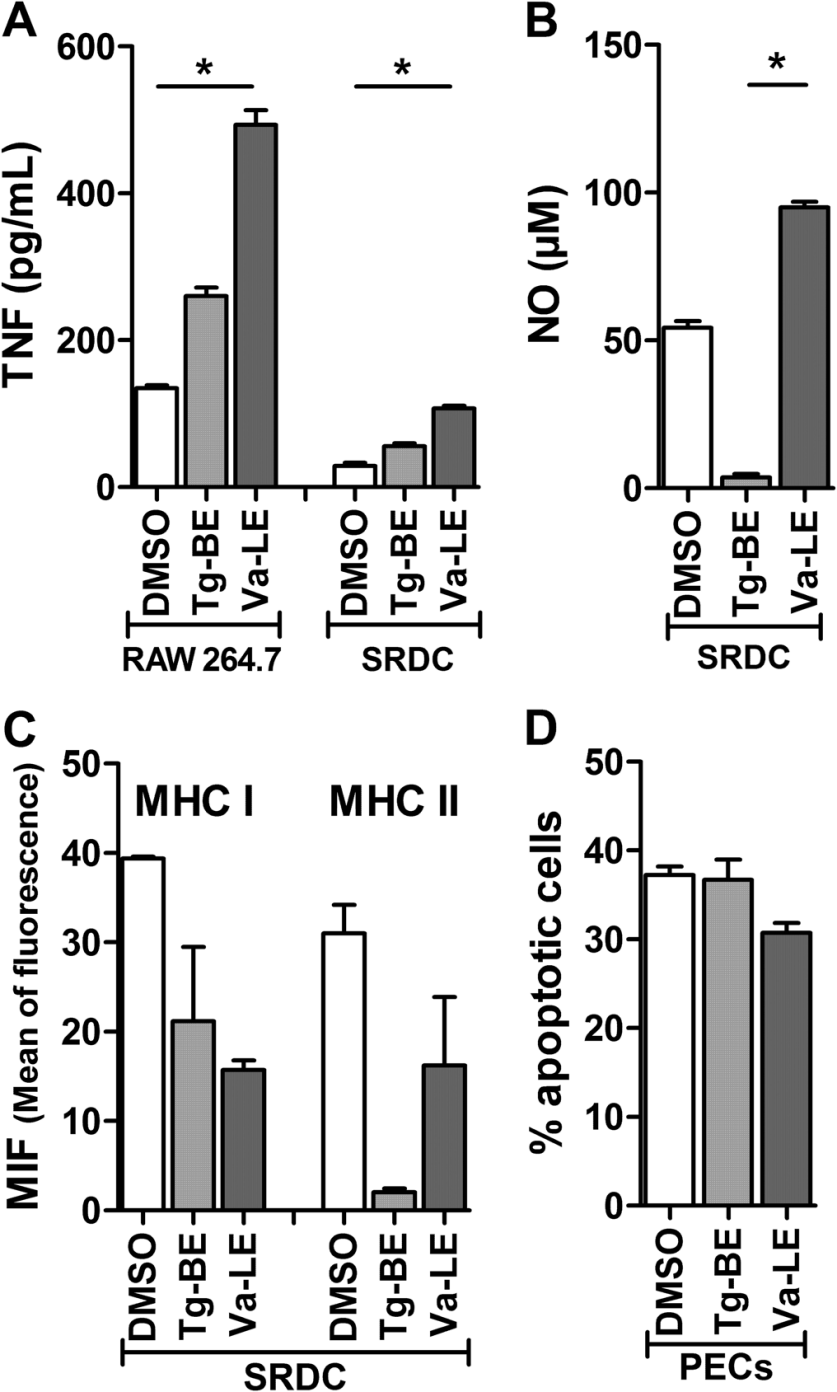
Cytokine production, MHC expression, and apoptosis of antigen-presenting cells. RAW 264.7 macrophages (A), SRDC dendritic cells (A, B, C), or peritoneal exudate cells (PECs) (D) were stimulated for 24 h with 10 μg/mL of Tg-BE or Va-LE, or with DMSO as negative control. TNF-α (A) and NO (B) were quantified in supernatant by sandwich ELISA and the Griess method, respectively (n = 3). Expression of MHC molecules of classes I and II was measured by flow cytometry after labelling with specific antibodies (n = 2). Apoptosis was evaluated by flow cytometry after labelling with annexin V-FITC and propidium iodide (n = 3). Results are mean ± S.D. *p < 0.05 (Dunn’s multiple comparison test).

We have observed that ethanolic extract of *T. grandis* bark and *V. amygdalina* leaves had the highest anti-*Toxoplasma* effect. An increase in TFN-α levels was measured in the serum of *P. berghei*-infected mice treated with ethanol extract of *V. amygdalina* leaves [26]. We thus aimed to determine whether the plant extracts could modulate responses of antigen-presenting cells like macrophages and dendritic cells. To answer this question, mouse macrophages of the RAW 264.7 cell line and mouse dendritic cells of the SRDC cell line were stimulated with 10 μg/mL of each plant extract. *V. amygdalina* but not *T. grandis* extract significantly increased the production of TNF-α by the two types of cells (Fig. 2A), and the production of NO by SRDC (Fig. 2B). In addition to cytokine production, an increase in the surface expression of MHC molecules of classes I and II is a marker of maturation of antigen-presenting cells. In contrast to what we expected, expression of both MHC molecules of classes I and II decreased on SRDC in response to Tg-BE and Va-LE. However, the differences with the cells stimulated with the solvent (DMSO) are not statistically significant (Fig. 2C). Polyphenols could be responsible for this decrease. Indeed, it has been shown that apple polyphenol extract decreased HLA-DR (MHC class II) expression on THP-1-derived human dendritic cells *in vitro* [14]. The authors suggested regulation through the ubiquitin-proteasome pathway. We have previously shown that glycosylphosphatidylinositols purified from *T. gondii* tachyzoites were able to increase MHC expression and antigen presentation on non-infected cells [7]. We suggested that their recognition and destruction as infected cells could be a strategy developed by the parasite to escape the immune system. *T. grandis* and *V. amygdalina* extracts may have immunomodulatory agents able to counteract the parasite effects on antigen-presenting cells.

Finally, we studied programmed cell death in response to the plant extracts. A recent review identifies the natural compounds able to target endoplasmic reticulum (ER) stress, modulated by inflammatory factors and resulting in apoptosis or autophagy [15]. These compounds include polyphenols, alkaloids, and saponins and some of them could be abundant in the plant extracts we tested here. Our results show that incubation of primary antigen presenting cells (PECs) with ethanolic extract of *T. grandis* bark or *V. amygdalina* leaves did not modulate the basal percentage of apoptotic cells (Fig. 2D). As primary cells, PECs are more susceptible to death than cell lines. Therefore, this last result comforts the low cytotoxic effect of these extracts observed on HFF.

## Concluding remarks

Plant extracts may act at different levels to produce anti-parasitic activities. Aside from direct inhibition of *T. gondii* proliferation, *T. grandis* and *V. amygdalina* extracts induced the production of TNF-α and NO, two pro-inflammatory molecules known to play an important role in resistance to *T. gondii in vivo*. The anti-oxidant activity of plant extracts is a parameter systematically studied. Our results indicated that the *V. amygdalina* extract with the highest parasite inhibitory effect had one of the lowest antioxidant activities. In the literature, anti-*T. gondii* activity was not related to antioxidant activity. For example, treatment with vanillin of *T. gondii*-infected mice induced a higher survival rate than treatment with resvan, a vanillin derivative with much higher anti-oxidant activity (19%, 85%, and 94% for vanillin, resvan, and ascorbic acid, respectively) [24]. Disappointingly, the direct effect of vanillin or resvan against *T. gondii* has not been investigated *in vitro*. However, the anti-oxidant properties could be important in the overall treatment of toxoplasmosis. Indeed, significant decreases in the total anti-oxidant ability were recorded 7 days after *T. gondii* infection in rats [4]. In *T. gondii* seropositive patients without symptoms, decreases of glutathione activity and increases of malondialdehyde related to lipid peroxidation reflected similar declines in the response to oxidative stress [13]. Dincel *et al*. have also shown that oxidative stress due to reactive oxygen/nitrogen species and NO production at pathological levels play a role in worsening neurodegeneration in the process of *Toxoplasma* encephalitis [9]. An anti-oxidant organoselenium compound reversed the alterations (splenomegaly, increase in reactive oxygen species levels and adenosine deaminase activity) found in the spleen of *T. gondii*-infected mice [10]. On the contrary, diet supplementation with vitamin E and selenium was associated with higher cyst numbers in the brains of infected mice [16]. Thus, strong antioxidants can inhibit the formation of free radicals involved in parasite resistance, leading to lower therapeutic efficacy. Markers of oxidative stress must be followed in case of infection with *T. gondii* and treatment with different plant extracts could be considered to obtain a balance between pro- and anti-oxidant factors in order to fight the parasite without deleterious side effects.

## Conflict of interest

The authors declare that they have no conflicts of interest in relation to this article.
